# How does air pollution affect urban settlement of the floating population in China? New evidence from a push-pull migration analysis

**DOI:** 10.1186/s12889-021-11711-x

**Published:** 2021-09-17

**Authors:** Zhihao Zhao, Xin Lao, Hengyu Gu, Hanchen Yu, Ping Lei

**Affiliations:** 1 School of Economics and Management, China University of Geosciences (Beijing), Beijing, China; 2 Department of Geography and Resource Management, The Chinese University of Hong Kong, Hong Kong, China; 3 School of Geographical Sciences and Urban Planning, Arizona State University, Tempe, AZ USA; 4 Key Laboratory of Carrying Capacity Assessment for Resource and Environment, Ministry of Natural Resources, Beijing, China

**Keywords:** Air pollution, Settlement intentions, Floating population, Heterogeneity, Push-pull migration model

## Abstract

**Background:**

Severe air pollution in China threatens human health, and its negative impact decreases the urban settlement intentions of migrants in destination cities. We establish a comprehensive framework based on the push-pull migration model to investigate this phenomenon.

**Methods:**

We employ a logistic model to analyze air pollution’s impact on the settlement intentions of the floating population based on the CMDS 2017 in China, combining the city-level socioeconomic variables with the individual-level variables.

**Results:**

Our results show that the annual average concentration of PM2.5 increases by 1 unit and that the probability of migrants’ settlement intentions will decrease by 8.7%. Using a heterogeneity analysis, we find that the following migrant groups are more sensitive to air pollution: males, people over 30 years old, less educated people, and migrants with nonagricultural *hukou*. With every 1 unit increase in PM2.5, each group’s settlement intentions decrease by 13.2, 16.7, 16.9, and 12.6%, respectively.

**Conclusions:**

Our results are consistent with existing studies. This study discovers that both external environment and internal factors influence migrants’ settlement intentions. Specifically, the differences in population sizes, economic development levels, public services, infrastructure conditions, and environmental regulations between cities play a significant role in migration decisions. We also confirm heterogeneous sensitivities to air pollution of different migrant subgroups in terms of individual characteristics, family factors, migration features, social and economic attributes.

**Supplementary Information:**

The online version contains supplementary material available at 10.1186/s12889-021-11711-x.

## Background

The population is the foundation of urban survival and the main driving force for creating wealth. In China, the floating population provide a large “demographic dividend” in urban wealth accumulation and economic innovation [1, 2]. This group is usually young and has a better educational background and higher mobility [3, 4]. They have high bargaining power and can actively move to their favourite areas [5]. With the increasing population and industrial agglomeration since reform and opening-up, air quality has become increasingly worse due to a long-term economic development pattern involving high pollution and high emissions [6, 7]. As smog has become more prevalent, it has also deeply affected people’s life behaviours [8–10]; this influence primarily manifests in increasing resident health costs and declining happiness [11–14]. As part of the urban population, the floating population also suffers from environmental change. This ecological change may cause them to leave the city.

The factors influencing this population’s settlement intentions in cities have received considerable scholarly attention. According to previous studies, regional features and demographic characteristics mainly affect the floating population’s destination selection decisions [3, 15–18]. Moreover, with promoting market opening and the loosening of China’s urban household registration management, people in China have been given more freedom to migrate among regions. When economic and public service needs are met, which seem to be primary concerns for rural-urban migrants, the environment plays a more critical role in migration decision making [19]. Studies have confirmed that when an origin area performs better than a destination area in terms of the ecological environment, the origin area’s pull force is more potent than that of the destination area, and the population will choose to leave the destination area [20, 21]. As one of the most easily perceived environmental factors, air pollution greatly influences the population’s immigration tendency. Especially in some regions with heavy pollution, haze negatively affects the long-term residential willingness of the floating population [22, 23]. This occurrence shows that a city’s air quality has become an important indicator that affects a city’s attractiveness, and it is closely related to people’s decisions to continue living in a city [4].

The willingness to settle in a destination reflects the floating population’s settlement behaviours. By investigating various factors in the destination city, migrants consider whether to live in the destination cities for a long time. There are many observations in research on the mechanisms driving migration, and the “push-pull” hypothesis is one of the most well-known explanations [24, 25]. This article is different from existing studies in the following points. First, we incorporate individual subjective factors into the theoretical model to analyse the impact of air pollution on the settlement intentions of the floating population. In the previous “push-pull” migration analysis, the individual’s subjective intention as a vital factor usually is ignored. Second, individual differences reflect different bargaining powers for the same environmental change. Floating population groups are characterized by different education levels, marital status, working background, etc. Due to differences in individual chracteristics, adaptability to the urban environment also shows varying systematic differences. In developing countries, economic development is still dependent on labour-intensive industries, and the floating population is the main source of labour in society [26]. Using these facts as a foundation, we assessed urban environmental governance’s specific impact on the labour market.

Based on data from a 2017 survey of China’s floating population, the study aims to explore environmental quality’s impact on settlement intention. A logistic regression approach is applied. Moreover, under the same air pollution exposure, we can distinguish different groups’ degree of sensitivity to air quality. This paper’s contributions can be demonstrated from the theoretical and practical perspectives. From the theoretical perspective, this study expands the application of the push-pull model, by using environmental changes as an entry point and combining the classic conceptual framework and individual settlement intentions to form migration potential, which can be used to find the impact indicators of population migration after China enters the new era. Furthermore, it confirms that air pollution, as an essential ecological risk, will affect migrants’ settlement intentions in China, and reveals the heterogeneity in the effects of air pollution on the urban settlement intentions among different sub-groups of migrants. From the practical perspective, the study’s results can provide evidence for formulating immigration management strategies, environmental regulation and urban governance for local governments.

The remainder rest of the paper is arranged as follows: The following section presents a literature review of relevant studies. Section 3 describes the research data and methods. Section 4 reports and analyzes the empirical results. Section 5 presents the heterogeneity test, and the final section presents the conclusion and discussion.

## Literature review and hypotheses

### Factors driving the settlement intentions of migrants

The term “floating population” is unique to China and is tied to the *hukou* system [27–29]. With the development of the economy and society, the floating population has gradually begun to settle in the cities where they work and to transfer their *hukou* to their new localities [29, 30]. The settlement intentions of the floating population in destination cities have aroused the concern of researchers.

Previous studies have suggested that the settlement intentions of floating populations are mainly affected by two factors: individual-level factors and regional-level factors. The floating population’s individual-level factors include personal attributes and socioeconomic elements [15, 31–33]. Among them, more attention is given to the population’s social integration level [28, 34–36], while economic conditions, such as job security, income level and housing conditions also affects migrants’ intentions to settle in destination cities [28, 30, 37]. Another category of determinants comes from the regional level, including economic, social, and policy factors. First, the floating population chooses to live in cities because the market mechanism facilitates their development [38, 39]. Second, policy factors are also a vital force driving migrants’ destination behaviours, especially the *hukou* system, which is tied to all aspects of people’s lives, affecting citizens’ social welfare [40]. Third, the social welfare system is related to residents’ living conditions [41]. By improving urban welfare coverage, migrant workers who enter cities are immediately included in the destination city’s social security system.

### Air pollution and migration

According to Tiebout’s (1956) “voting with their feet” theory, due to competition between local governments to provide public goods and voters’ freedom to migrate, residents migrate to maximize personal interests and balance marginal costs and benefits, which leads to Pareto improvement in the spatial economy [42, 43]. As a public product in the city that is inseparable from residents’ lives, urban air quality plays an increasingly prominent role in the urbanization process, workforce migration and settlement intentions [44, 45]. Severe smog intensifies immigrants’ perceived health risks and has negatively impacted their work location and migration decisions [19, 46–48].

Air pollution, a global environmental crisis, also affects China’s floating population regarding in-migration, living costs, and social inequality. First, air pollution significantly decreases migrants’ in-migration [4, 49]. Because of the direct health risks caused by smog, migrants choose to “escape” from heavily polluted cities [21, 50, 51]. Second, the ever-increasing smog has increased the floating population’s cost of living. Zhang and Mu (2017) confirmed that when a city’s air quality index (AQI) increases by 100 points, the consumption of anti-PM2.5 masks increases by 70.6%, which means that the public must spend a considerable amount of money to avoid pollution [52]. Third, Sun et al. (2017) found that smog also objectively exacerbates urban wealth differentiation and social inequality [53].

In summary, environmental factors have played a more critical role in migration’s influencing mechanism [34]. Air pollution hinders the floating population from living in the cities where they work and strengthens their willingness to return home to obtain clean air for their health [4, 49, 54]. However, literature focusing on the relationship between air pollution and migration in Chinese cities is currently rare. Based on the extant studies, the following hypotheses are proposed:

- H1: Air pollution affects population migration. As the air pollution level increases, the floating population’s willingness to stay will decrease accordingly.
- H2: The difference in air quality between the origin area and the destination area will impact migration. The larger the gap is, the more likely population migration is to occur.
- H3: Different groups within the floating population have different sensitivity levels to air pollution and will have different migration options.

## Data and method

The study used the 2017 China Migrants Dynamic Survey (CMDS) conducted in August 2017, which reflected the floating population’s migration status in 2017 and was published by China Migration Population Service Center (https://www.chinaldrk.org.cn). Questionnaires were used to complete a sample survey of the floating population in 31 provinces (regions, cities) in China in 2017. The survey is a nonrevisited sampling survey and cannot guarantee the sample’s continuity, so it cannot form panel data for research. The survey data cover migrants’ demographic characteristics, family status, settlement intentions, health and social integration. We eliminated the samples whose origin and destination were the same, and that did not include migration. Ultimately, 116,283 samples were retained. The statistical data for prefecture-level cities in 2016 were obtained from the “China City Statistical Yearbook 2017” and the “China City Construction Statistical Yearbook 2017”. The air quality data used in the article are the average PM2.5 of each city in 2016 and come from Global Annual PM2.5 Grids from MODIS, MISR and SeaWiFS Aerosol Optical Depth (AOD) with GWR.

The push-pull migration model emphasizes that the population can improve its quality of life through migration behaviour premised on freedom of movement [55]. The theory suggests that decisions between cities are based on comparing various factors between regions for the floating population. These factors encompass different levels of socioeconomic factors, environmental factors, political factors, and public service levels [2, 56–60]. They are divided into positive and negative factors according to whether they are conducive to population migration and constitute the “push” and “pull” of population migration. The push-pull migration model is also suitable for this study, for the pull force is the factors to improve the settlement intention mainly from the destination city, and the push force is the factors to decrease the settlement intention mainly from the origin city. This model is also combined with individual-level factors to raise its explanatory ability. Based on the classical push-pull theory, we built our regression model as follows:

1 $$ {Staywilling}_{ijt}={b}_0+{b}_1 pm{2.5}_{j,t-1}+{b}_2{city}_{j,t-1}+{b}_3{individual}_{it}+\varepsilon $$

In eq. (1), the subscript *i* is the floating population individual; *j* is the prefecture-level city of destination; and *t* is the year. The explained variable *staywilling*_*ijt*_ is a binary categorical variable: *staywilling*_*ijt*_ = 1 indicates that the floating population is willing to stay in the destination city for a long time in the future, and *staywilling*_*ijt*_ = 0 indicates that they are unwilling to stay. *Pm2.5*_*j,t-1*_ represents the average PM2.5 concentration of *city*_*j*_ in year_t-1_, and *city*_*j,t-1*_ is the city-level control variable, and individual_it_ is the individual-level control variable for the floating population. Because immigration behaviour has a certain time lag, the city’s floating population after a certain number of years of life before making immigration decisions. The city-level variables all lag behind the dependent variable by 1 year, thus relieving the endogeity issue to some extent. Moreover, the dependent variable we use is the settlement intentions of the floating population. As a subjective self-assessment, it truly reflects migrants’ self-assessment of living quality in destination cities, which motivates them to choose to stay in or leave the destination area for an extended time. The independent variable uses the PM2.5 difference between the destination and origin areas to reflect the impact of air pollution on the settlement willingness. However, as a person’s subjective intentions, settlement intention will not practically impact the objective air pollution level. It will only reflect PM2.5’s actual impact on the settlement intentions of floating population in cities. This impact will affect future migration decisions and is a one-way relationship. Therefore, we believe that in this exploration process, no strong endogeneity will occur.

And the classical gravity model can be expressed as [61]:

2 $$ {M}_{ij}=k\frac{P_i^{b_1}{P}_j^{b_2}}{d_{ij}^c} $$

In eq. (2), *M*_*ij*_ means the number of immigrants between areas *i* and *j*. *P*_*i*_ and *P*_*j*_ mean the influence factors of the city i and j. And *d*_*ij*_ represents the distance from the city *i* to *j*. The equation shows that immigration activities are simultaneously affected by the destination and the origin place, and the push and pull forces between the two determine the final number of immigrants under the influence of geographic distance. Combining Eq. (1) and (2), the conceptual model of this study can be represented by:

3 $$ {Staywilling}_{nijt}={b}_0+{b}_1\frac{pm{2.5}_{j,t-1}}{pm{2.5}_{i,t-1}}+{b}_2\frac{city_{j,t-1}}{city_{i,t-1}}+{b}_3{individual}_{nt}+\varepsilon $$

In eq. (3), the subscript *n* is the floating population individual; *j* is the destination city, and *i* is the origin city. The explanatory variable selected for the study is the floating population’s willingness to stay for a long time. It is constructed as a binary categorical variable (“willing to stay” as 1 and “unwilling to stay” as 0). The explanatory variable is the annual average concentration of PM2.5, with the prefecture-level city as the spatial unit, signifying the natural environmental conditions. As the smog’s core pollutant, PM2.5 usually be used to indicate the quality of the regional environment [14, 49, 62]. By calculating the average concentration ratio in the origin and destination cities, it can be observed whether the difference in PM2.5 concentration between the two cities exerts a “pull” or “push” force on migrants’ destination decisions. The control variables for the study are selected from two levels: city and individual. We constructed categorical variables for some of the control variables according to the data type, and the processing methods are shown in Table 1.

**Table 1:** Preprocessing of control variables

Variables		Processing	
Panal A: City level			
Lnpgdp		Logarithm of GDP per capita	
Unemployment		Unemployment rate	
Undergraduate		Number of college students per 10,000 people	
Pop		City population at the end of the year	
Perroad		Road area per capita	
Rubbish		The decontamination rate of urban refuse	
Migrate		1-East to east	
		2-Mid-west to east	
		3-East to mid-west	
		4-Mid-west to mid-west	
Panal B: Individual level			
Education	1-Primary and below	Status	1-Employee
	2-Junior school		2-Employer
	3-Senior school		3-Self-employment
	4-college and above		4-Others
Marriage	0-Unmarried	Income	1-Less than 4200
	1-Married		2–4200-6000
Family	1-Single		3–6000-9000
	2-Spouse		4–9000 and above
	3-Child	House	1-Free housing
	4-Parents		2-Tenement
	4-Others		3-Self-buying
*Hukou*	0-Urban	Medical insurance	0-No
	1-Rural		1-Yes
Range	0-Inter-provincial	Social security card	0-No
	1-Intra-provincial		1-Yes
Duration	Duration of this migration		

**Fig. 1:**
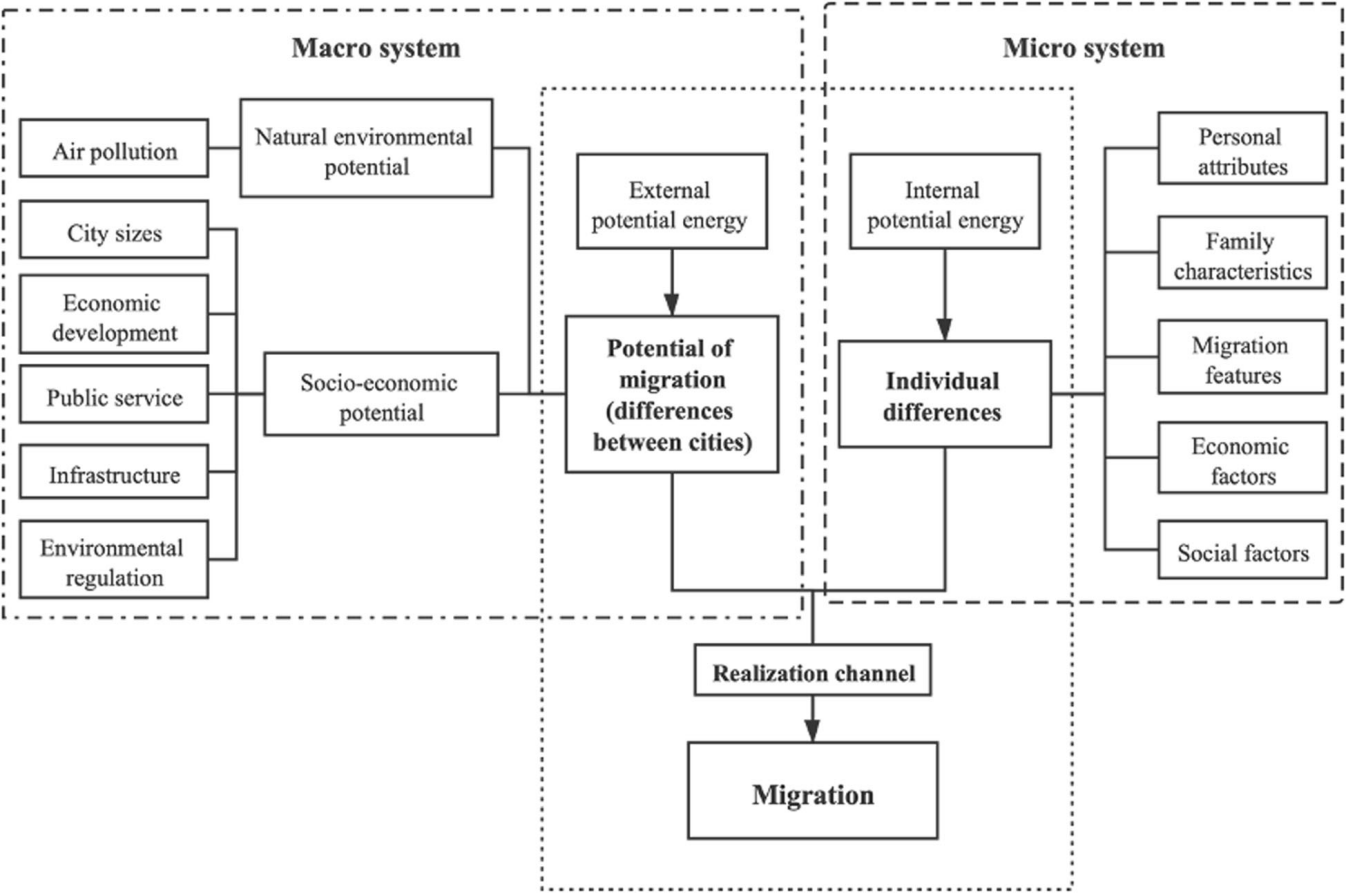
The potential energy conversion of migration framework

Based on the classical push-pull migration theory proposed by Lee (1966), we constructed the conceptual framework of this study [55]. The differences between the destination and the origin cities generate external potential energy for migration, and the characteristics of the floating population also generate internal potential energy for migration. Under the combined effect of these forces and the migration channel’s intermediate variable, the floating population’s settlement intentions change (the mechanism is shown in Fig. 1).

First, the differences between cities constitute the external variable of population migration—potential external forces. At the macro level, the natural environment and socioeconomic differences between different regions provide personal development opportunities for the floating population, which creates an external driving force. These differences are as follows: (1) City sizes: large cities offer more resources that attract the floating population [63, 64]. (2) Economic development: the population prefers to migrate from economically underdeveloped cities to economically developed cities [26, 56]. (3) Public service: a complete social care system is also an external potential that attracts the floating population to a city [33, 65]. (4) Infrastructure conditions: The per capita road area can reflect the urban traffic situation. The larger the average road area per person, the more convenient the traffic situation may be. (5) Environmental regulation: studies have shown that a clean urban environment creates external potential energy, stimulating the floating population.

Second, from the micro perspective, internal variables create the internal potential energy that drives migration. Therefore, we selected the individual-level factors containing personal attributes, family characteristics, migration features, economic factors, and the floating population’s social factors. The control variables are as follows: (1) Personal attributes: Both age and gender are basic factors of the floating population [66]. Additionally, marital status connects individuals to formed families [67]. (2) Family characteristics: the family factors of the floating population in the migration process. (3) Migration features: the scope and duration of the migrant population’s mobility [68]. As a household registration management system specific to China, the *hukou* system has a highly restrictive effect on population movement [29]. (4) Economic factors: the current economic situation directly related to occupation and income [69]. (5) Social factors: basic social security such as medical care and housing reflects the satisfaction of the floating population’s basic living needs [70].

Third, the channel through which migration is realized is an important intermediate variable. A series of intervening obstacles are found between the destination and origin areas in the actual migration process, preventing the floating population’s free migration. Migration distance is reflected in interprovincial migration and intraprovincial/intercity migration. If the distance is long, people will consider local conditions more carefully and have more stringent air quality requirements [49].

Tables 2 and 3 show the descriptive statistics for the continuous variables and categorical variables, respectively, in the study. To prevent multicollinearity between variables, we conducted a collinearity test, and the result indicates no multicollinearity exists between variables.

**Table 2:** Descriptive statistics of continuous variables

Variable	Description	N	Mean	Std.Dev.	Min	Max
Staywilling	The willingness of long-term stay	116,283	0.829	0.377	0	1
PM2.5	PM2.5 annual average ratio	116,283	1.032	0.405	0.138	5.80
Lnpgdp	Per capita GDP ratio in logarithmic form	116,283	1.074	0.41	0.027	3.78
Unemployment	Ratio of unemployment rate	116,283	1.138	1.32	0.021	38.08
Undergraduate	The ratio of the number of college students per 10,000 people	116,283	7.232	18.12	0.001	1027.12
Pop	The ratio of the number of medical beds per 10,000 people	116,283	8.016	13.153	0.010	230.67
Perroad	Per capita road area ratio	116,283	1.017	0.67	0.096	9.12
Rubbish	The decontamination rate of urban refuse	116,283	1.075	0.307	0.197	5.09
Duration	Duration of this flow	116,283	6.017	5.99	0	69

**Table 3:** Descriptive statistics of categorical variables

Variables	Description	Categories	Freq.	Percent
Migrate	City location from origin to destination	1-East to East	26,451	22.75
		2-Midwest to East	37,519	32.27
		3-East to Midwest	5023	4.32
		4-Midwest to Midwest	47,290	40.67
Education	Individual education level	1-Less than Primary	17,966	15.45
		2-Junior school	51,137	43.98
		3-Senior school	25,501	21.93
		4-College above	21,679	18.64
Marriage	Individual marital status	0-Unmarried	21,545	18.53
		1-Married	94,738	81.47
*Hukou*	*Hukou*	0-Rural	25,919	22.29
		1-Urban	90,364	77.71
Range	The range of flow	0-Cross-provincial	71,177	61.21
		1-Cross-city in the province	45,106	38.79
Status	Employment status	1-Employee	59,592	51.25
		2-Employer	5632	4.84
		3-Self-employment	31,159	26.80
		4-Others	19,900	17.11
Income	Monthly household income	1-Less than 4200	28,854	24.81
		2–4200-6000	21,214	18.24
		3–6000-9000	35,934	30.90
		4-Above 9000	30,281	26.04
House	Family housing	1-Free housing	17,098	14.70
		2-Tenement	69,883	60.10
		3-Self-buying	29,302	25.20
Medical insurance	Whether they have medical insurance	0-No	9981	8.58
		1-Yes	106,302	91.42
Social security card	Whether they have a social security card	0-No	56,136	48.28
		1-Yes	60,147	51.72
Family	Number of accompanied family members	1-Single	13,186	11.34
		2-Spouse	94,738	81.47
		3-Child	1488	1.28
		4-Parents	6871	5.91

## Empirical results

### Estimates from the regression model

Table 4 reports the basic regression results, with Model 1 being the result of controlling the floating population’s individual characteristics. Air pollution negatively impacts the floating population’s settlement intentions, but it is not significant. Model 2 results from controlling city-level variables. The regression coefficient of the PM2.5 gap is − 0.081 and significant at the 1% level. If the coefficient is negative, the possibility that the explained variable takes the value of 1 is less. In short, the more serious the air pollution is, the lower the willingness to stay. Finally, model 3 includes variables for both individual and city characteristics, and the original results hold. Interpreted from the log odds ratio perspective, the log odds ratio of the floating population choosing to leave will increase by 91 (e^-0.087^) times for each additional unit of the PM2.5 gap between cities. This result is significant at the 1% level and confirms Hypothesis 1: the floating population will “vote with their feet” to respond to air pollution [43].

**Table 4:** Results from logistic regression

Dependent variables	Model1	Model2	Model3
Staywilling	(1)	(2)	(3)
PM2.5	−0.038 (− 1.94)	−0.081^***^ (− 4.07)	−0.087^***^ (− 4.25)
Lnpgdp		0.084^***^ (3.99)	0.189^***^ (8.63)
Unemployment		−0.018^**^ (− 3.03)	− 0.026^***^ (− 4.43)
Undergraduate		0.011^*^ (2.48)	0.0004 (1.05)
Pop		0.012^***^ (14.08)	0.008^***^ (9.04)
Perroad		−0.102^***^ (−8.63)	− 0.085^***^ (−6.94)
Rubbish		0.140^***^ (5.16)	0.115^***^ (4.17)
Direction (Reference: East to East)			
Midwest to East		−0.386^***^ (−16.36)	− 0.135^***^ (−4.96)
East to Midwest		−0.508^***^ (−12.84)	−0.364^***^ (−8.57)
Midwest to Midwest		−0.352^***^ (−15.93)	− 0.210^***^ (−9.06)
Education (Reference: Less than primary)			
Junior school	0.293^***^ (13.39)		0.284^***^ (12.93)
Senior school	0.467^***^ (17.60)		0.456^***^ (17.06)
College and above	0.710^***^ (22.13)		0.696^***^ (21.46)
Marriage (Ref: Unmarried)			
Married	0.371^***^ (14.25)		0.393^***^ (15.03)
*Hukou* (Ref: Agricultural)			
Non-agricultural	−0.092^***^ (− 4.13)		− 0.099^***^ (−4.37)
Range (Ref: Inter-provincial)			
Intra-provinicial	0.049^**^ (2.89)		0.085^***^ (3.99)
Duration	0.031^***^ (20.17)		0.029^***^ (18.83)
Status (Reference: Employee)			
Employer	0.097^*^ (2.12)		0.181^***^ (3.93)
Self-employment	−0.169^***^ (−8.50)		− 0.091^***^ (−4.42)
Others	− 0.051^*^ (−2.13)		− 0.015 (− 0.61)
Income (Reference: 9000 and above)			
Less than 4200	− 0.635^***^ (−24.63)		− 0.546^***^ (− 20.82)
4200–6000	− 0.470^***^ (−17.71)		−0.405^***^ (−15.10)
6000–9000	− 0.274^***^ (− 11.21)		− 0.239^***^ (− 9.73)
Medical insurance	0.062^**^ (2.21)		0.138^***^ (6.52)
Social security card	0.122^***^ (7.23)		0.097^***^ (5.61)
House (Reference: Free housing)			
Rent	0.119^***^ (5.44)		0.102^***^ (4.63)
Self-buying	0.774^***^ (25.54)		0.809^***^ (26.52)
Family (Reference: Single)			
Spouse	0.500^***^ (6.63)		0.527^***^ (6.97)
Child	0.060 (1.35)		0.133^***^ (2.98)
Parents	0.244^***^ (4.08)		0.260^***^ (4.33)
Constant	0.913^***^ (16.69)	1.750^***^ (41.01)	0.797^***^ (10.49)
Pseudo R^2^	0.05	0.01	0.06
Log likelihood	−50,598.39	−52,747.26	−50,279.64
N	116,283	116,283	116,283

t statistics in parentheses

* p < 0.05, ** p < 0.01, *** p < 0.001

#### Influencing factors at the macro-level

We found that when PM2.5, a major particle of smog, has a higher concentration level in the destination area than in the origin area, it is less likely that migrants will stay for long periods. For the floating population, the cities in which they once lived serve as a reference for comparison, and the more significant the gap between smog’s severity in the current city and the past city is, the more it will make migrants unhappy, which in turn will affect their destination choices [49]. Analyzing the regression results of various city-level variables yields the following conclusions.

First, a city’s economic development level directly affects the migration decision of the floating population. The GDP per capita is directly related to its settlement intentions, proving that the greater the difference in economic development between cities, the more significant the attraction effect on the floating population is. Developed cities exert a “pull” force on migrants, aligning with existing conclusions [68]. Economic development remains the most critical factor driving migration, as reflected in the results regarding the unemployment ratio. As the unemployment rate gap between cities increases, migrants may face more significant employment pressures and more occupational instability, which may then cause them to make decisions to move on or return home without remaining in the city for extended periods.

Second, education in cities has external potential energy on the floating population’s settlement, consistent with prior studies’ results [65, 66]. Studies have shown that the aggregation effect of highly educated people in an urban area provides a notable human capital and innovation advantage for urban development and creates more jobs. Increased employment opportunities motivate long-term settlement of the floating population in search of better personal development potential.

Third, cities’ infrastructure can also influence the floating population’s settlement intentions [71]. The regression results show that the greater the per capita road area is, the less it will attract migrants as a long-term destination. It may be that the mere increase in road area does not improve urban traffic problems. This supposition aligns with the “Braess Paradox”, where expanding the urban transport network results in more severe traffic congestion [72].

In summary, the differences between cities produce different external energy potentials for the migration of the floating population, and this result validates Hypothesis 2.

#### The influencing factors at the micro-level

Population migration is influenced by city characteristics and site selection decisions based on individual limitations. We controlled for individual characteristics, namely, the education level, marital status, register, floating characteristics, occupation and public service factors of migrants. It can be seen from Model 3 in Table 4 shows that the floating population’s *hukou* significantly impacts settlement intentions. Compared to the floating population with urban *hukou*, the floating population with rural hukou is less likely to reside in cities [73]. The local complex is an integral part of traditional Chinese culture. For Chinese people, the hometown plays a significant role in life. Especially for older people with rural *hukou*, the impact is quite profound, and they do not choose to stay in the destination location.

Regarding education, the floating population with a higher education level has more knowledge and skills and has a more obvious advantage in the job market. The more years of education a migrant has, the better job they have, and the stronger their willingness to stay in the resident city [12]. Family factors are also key issues that migrants must consider when making migration decisions. Marriage is the foundation of the family. Among the floating population, those who are married have relatively fixed family bonds, making the individual’s willingness to migrate more subject to family relations. If this group chooses to “vote with their feet”, they must give up the benefits they have already gained in the inflow area and pay high migration costs. Since this is likely an uneconomic choice, so they always choose to continue living in the city [74].

Table 4 also reports results on the income and the level of individual social security. The migrants who enjoy medical security, social security, high income and housing guarantees have achieved basic security and resolved certain concerns, which increase their willingness to continue living in the city.

### Robustness tests

To test the results’ accuracy, we conducted robustness tests. The robustness of two different testing methods was investigated by replacing the independent variables with the AQI and replacing the regression model with the probit regression model. Compares to PM2.5, the AQI is a comprehensive index used to describe the degree of air cleanliness and closely related to residents’ health. Thus, we use AQI instead of PM2.5 for the regression. Column (1) of Table 5 reports the AQI regression results; the regression coefficient is − 0.130, significant at the 1% level. The two most commonly used binary selection models are the logit model and the maximum likelihood estimation probit model. To further test the validity of our results, we replaced the empirical model and used the probit model to regress air pollution and the willingness to stay of the floating population. The results are reported in column (2) of Table 5, and the estimated coefficients are consistent with the benchmark model. The results of the two robustness tests indicate that the regression results are reliable.

**Table 5:** Results from robust tests

Dependent variables	(1)	(2)
Staywilling		
AQI	− 0.130^***^ (− 4.50)	
PM2.5		−0.049^***^ (− 4.32)
Lnpgdp	0.191^***^ (8.41))	0.110^***^ (8.78)
Perroad	−0.085^***^ (−6.91)	− 0.050^***^ (− 7.18)
Rubbish	0.112^***^ (3.77)	0.062^***^ (3.87)
Education (Reference: Less than primary)		
Junior school	0.288^***^ (12.98)	0.165^***^ (12.98)
Senior school	0.461^***^ (17.23)	0.265^***^ (17.52)
College above	0.703^***^ (21.65)	0.392^***^ (21.89)
*Hukou* (Ref: Agricultural)		
Non-agricultural	−0.090^***^ (−4.03)	−0.047^***^ (−3.82)
Status (Reference: Employee)		
Employer	0.174^***^ (3.76)	0.084^***^ (3.44)
Self-employment	−0.097^**^ (−4.73)	− 0.057^***^ (− 4.90)
Others	− 0.022 (− 0.94)	−0.012 (− 0.89)
City level	Yes	Yes
Individual level	Yes	Yes
Constant	0.788^***^ (10.95)	0.458^***^ (11.86)
Pseudo R^2^	0.055	0.056
vLog likelihood	−50,296.77	−50,284.60
N	116,283	116,283

t statistics in parentheses

* p < 0.05, ** p < 0.01, *** p < 0.001

Due to space restrictions, only the results of key variables are reported in the table.

## Further exploration: heterogeneity analysis

**Fig. 2:**
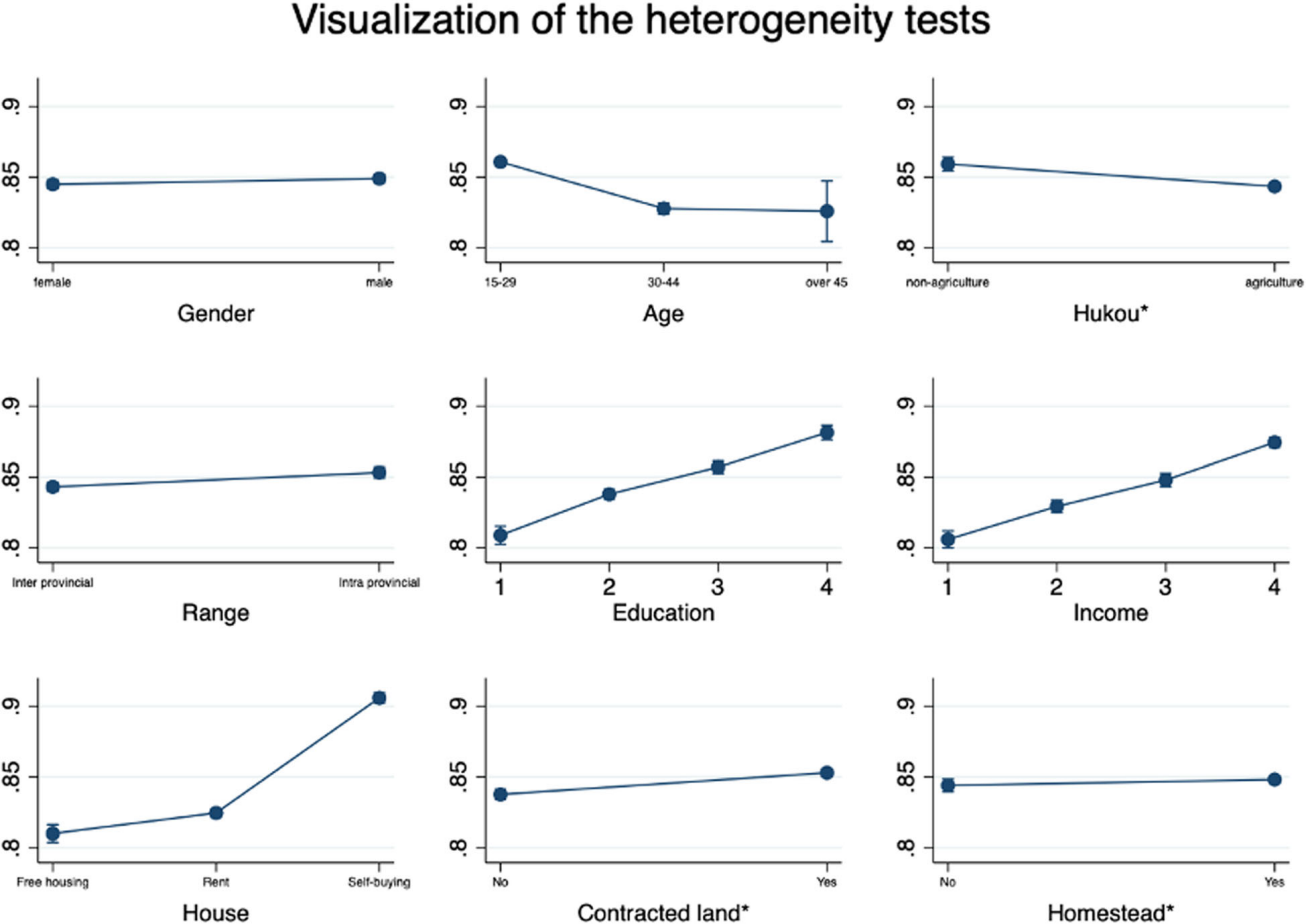
Visualization of the heterogeneity analysis. Notes: The Y-axis represents the marginal effect of the intentions to stay in the destination city. *Hukou: The household registration management system specific to China, which divided into “non-agriculture” and “agriculture”. *Contracted land and Homestead: rural contracted land and rural homesteads are important land factors that facilitate the rural population’s agricultural production activities and family housing security

Individuals in the floating population have different characteristics. This section discusses the degree of smog sensitivity of different floating populations and their heterogeneous effects on settlement intentions. We conducted regressions on the sample groups from different angles. The regression model used is the same as Model 2 above. The regression results in Additional file 1 of this paper. We visualised the marginal effect results to more clearly display the results, as shown in Figure 2.

### Gender

First, the research samples are grouped according to gender, which shows that the more serious the air pollution in the destination city, the less willing women and men are to settle there. However, the male migrant population is more sensitive to air pollution, and the negative effect of PM2.5 on the male population’s willingness to stay is more significant. When the concentration of PM2.5 in a city’s air increases by 1 μg, men’s settlement intentions will drop by 13.2%., which may result from migrant men’s occupational and life characteristics. Men are more likely than females to be exposed to air pollution and exposed to greater levels of automobile exhaust and pollution from industrial equipment [31].

### Age

Age is an essential demographic feature [32]. We divided the samples into three age groups: 15–29, 30–44, and 45 and above. Migrants aged 15–29 are not highly sensitive to air pollution and PM2.5. Also, their willingness to stay did not have the expected negative effects. In the 30–44 and over 45 groups, PM2.5 has negatively affected settlement intentions. Such results may be explained as follows: (1) In the population migration process, young people tend to pay more attention to employment opportunities. Economic factors play a decisive role at this stage, and younger migrants may even choose to ignore air quality to improve their income. (2) As the floating population enters the middle-aged and elderly phases of life, these individuals usually have accumulated certain economic benefits, and their demands for physical health and quality of life increase, which means that they increasingly prefer cleaner air [75]. This was also described by Sun et al. (2019).

### Educational background

Figure 2 also visualizes migrants by group according to their different educational backgrounds. For the floating population groups with junior college degrees or below, PM2.5 significantly and negatively affects their settlement intentions. Among them, senior high school graduates are the most sensitive to smog, and their sensitivity is higher than those with primary and junior high school diplomas. Completely different results are found for the floating population with a college degree and above. The higher the PM2.5, the more likely this group is to stay in the city. It may be that people with a high degree of education have more knowledge and skills that enable them to live in cities, and the compensation they receive in the labour market is high. They can reap higher returns when they move to the city for employment and development, and the economic benefits are more significant than the cost of air quality improvement, which means that they will more likely choose to stay [33].

### “*Hukou*” and migration distance

China’s *hukou* system is the main policy factor restricting the settlement of migrants in cities [76]. We carried out a group regression to explore the attitudinal differences between people with nonagricultural and agricultural *hukou*. For nonagricultural migrants, PM2.5 does not affect their settlement intentions. For the group with agricultural *hukou*, air pollution has a significant negative impacts on their settlement. The nonagricultural floating population is less limited by the difference between urban and rural *hukou* during the migration process. The cost of accommodation in the local city is relatively small, which reduces the impact of air quality’s impact on their destination choice [15].

For the different migration ranges, compared with that of migrants within a province, the willingness of interprovincial migrants to stay in the city is more negatively affected by smog. The farther the migration distance is, the higher the migration cost. In China, the cultural tradition of being attached to one’s native land still has influence and makes people unwilling to leave their hometown. Therefore, different attitudes toward choosing a place to live between the interprovincial migrants and interprovincial migrants exist. For prefecture-level cities under the same province’s jurisdiction, the shorter geographic distance and more remarkable lifestyle similarity make intercity in the same province migrants less sensitive to air pollution.

### Household monthly income

Income is the basis of the floating population’s livelihood. Using the floating population’s family income in the CMDS data, we constructed dummy variables corresponding to four different income levels for group regression, and the results are shown in Fig. 2. In general, the higher the income, the greater the sensitivity to air pollution is. This is because families with a low-income focus more on their current work, as they need to maintain their ability to meet their daily living expenses [39]. For them, the cost of moving to other cities exceeds their income, and migration is not worth the expense. When the family’s monthly income exceeds 4000 yuan, they have the necessary funds to improve their lives. When the cost of air pollution to the floating population exceeds the current benefits of residing in the contemporary city, they will most likely choose to “vote with their feet” and leave. In other words, families with a monthly income of more than 4000 yuan are more sensitive to air pollution, and the air pollution of the city they currently live in will create a greater push to drive them away.

### Housing situation

In this section, we group and regress the research samples based on the three types of housing conditions, “free housing” (housing provided by the company, etc.), “self-rental housing”, and “self-purchased housing”, to explore the heterogeneity under different housing conditions. The results show that the first two groups’ settlement intentions are significantly and negatively affected by air pollution, while the self-purchased housing group’s settlement intentions are not. The reason is that the first two groups’ housing needs have a short-term effect on their settlement intentions. However, urban air pollution control cannot be completely reversed in the short term. Therefore, to meet their long-term needs for better air quality in the future, they are better positioned to move. However, considering property disposal and family burdens, the self-purchase group is less sensitive to air pollution than the first two groups.

### Rural contracted land and homesteads

In China, rural contracted land and rural homesteads are important land factors that facilitate the rural population’s agricultural production activities and family housing security [77]. We grouped the sample according to these two land factors and explored air quality’s impact on the floating population’s settlement intentions under different rural land occupation statuses. For the floating population, regardless of whether they have contracted land, air pollution has a significant negative effect on their willingness to stay, and little difference exists between the two groups. This situation also appears in the homestead heterogeneity test, which did not show significant intergroup differences. The homestead is an important safeguard of the floating population’s livelihood. When the floating population becomes disgusted with the air pollution in their city, they can choose to return to their hometown [78].

Overall, the floating population responds to smog in different ways according to individual characteristics. This confirms Hypothesis 3 of this study.

## Conclusions and discussion

As severe air pollution has emerged as an issue in China, urban residents have expressed a growing desire for clean air. After controlling for regional-level factors and the individual factors of migrants, this study found that air pollution has considerably impacted population migration. In the face of urban air pollution, the floating population will “vote with their feet” (Banzhaf and Walsh,2008), moving from places with severe air pollution to locations with good air quality. Each time the annual average PM2.5 level increases by 1 unit, the probability that the floating population will choose to settle in the destination city for an extended time drops by 8.7 percentage points, demonstrating a willingness to sacrifice income for environmental quality. On average, the floating population’s willingness to pay for a PM2.5 concentration reduction of 1 μg/m3 is approximately 1034.08 yuan (calculated based on the national per capita disposable income in 2016). This result is similar to the calculation result reported in prior research [4, 49, 79]. As a vital indicator of the settlement inntentions of floating population, environmental quality plays a more and more significant role in the future. We incorporated it into the classical “push-pull” migration model, confirming the negative effect of air pollution on the migration intentions. The results prove that it is inevitable for the floating population to consider air quality in migration decisions. This also enlightens future research on immigration influencing factors, which should pay attention to the exogenous effects of urban environmental conditions.

Second, the difference between the origin and destination cities in terms of urban economic development, education level, public service level, and air pollution will push and pull the floating population’s migration [68]. Moreover, the larger the gap is, the less likely the floating population will be willing to stay in the destination city for an extended time. Severe air pollution has caused a decline in the floating population’s willingness to stay in the destination city, which indicates that the floating population may choose to leave the city with increasing PM2.5 concentration in the future. This immediate environmental risk impacts the future supply of urban labour. Although this negative effect may have hysteresis, it drives cities to pay attention to pollution hazards. As a major contributor to environmental pollution, PM2.5 closely relates to the level of urban development and urban traffic conditions [13]. When the cost of living caused by air pollution exceeds the city’s economic benefits, people are more likely to “vote with their feet” and leave the residential areas, which verifies the existence of “environmental migration” in China [4]. With the improvement in the living standards, immigrant groups have also increased their demands for a living environment, and paid greater attention to environmental quality’s important role in urban amenities during the immigration process [20].

Third, different floating population’s sensitivity to air pollution is heterogeneous. Specifically, migrants who are males, 30 years of age or older, less educated, and have nonagricultural *hukou* are more sensitive to air pollution. However, migrants whose migration path is interprovincial, who have no private property, and whose monthly household income is more than 4000 yuan are more affected by air pollution. We also found that the rural land factor is an important influencing factor for China’s floating population. Rural migrants who have homesteads in their hometowns are sensitive to air pollution in their destination cities. Clean air in cities is a public good that everyone needs. However, different groups have different bargaining powers [80]. In the process of obtaining public goods, there are differences in the results of competition according to different economic status. In order to maintain the sustainability of society, it is necessary to take into account the common needs of different groups for clean air. This requires governments to implement effective environmental regulations to ensure the realization of environmental justice. It also enlightens researchers on considering group heterogeneity in the studies on the effect of air pollution on migration.

This study’s results have specific policy implications. First, with the ageing of the population intensifying and the number of labourers declining, this study’s conclusions have great significance in the current battle for talent raging among cities. After China’s transition from emphasizing the economic growth pace to the economic development quality, the ecological environment has become an important part of green development. As a result, physical health and urban environment quality influence the migration of talent. Therefore, urban amenities have become an essential element of competition between cities [29]. Cities can attract more talent by implementing strict air quality standards, limiting automobile exhaust emissions, strengthening coordinated environmental governance, and creating livable cities. Second, the economic and social development gap between destination and origin cities contributes to the push and pull of population migration. As cities that receive a large inflow of migrants, developed cities in the east should speed up the floating population’s *hukou* transfer process and equalize public services [15, 76]. As cities that see the largest outflow of migrants, small cities in Central and Western China should develop clean industries to realize economic gains. Through industrial upgrade and public services improvement, the floating population can be pulled back to their hometowns. When underdeveloped regions undertake polluting industries transferred from developed regions, they should weigh the relationship between pollution control and economic development. Third, targeted policies for the floating population should be strengthened. Through vocational training, the employment quality of migrant workers can be improved [36, 70]. In addition, in the context of rural land reform, the urbanization of farmers can be promoted through the transfer of homesteads, which will further promote China’s new type of urbanization development.

Although we have considered the individual level and regional-level variables that influence the floating population as comprehensively as possible and have analyzed the sensitivity of different floating populations to air pollution, this study still has some limitations. Limited by the availability of data, this study uses cross-sectional data from 2017. It is challenging to analyze air pollution’s effect on the willingness to stay in the time dimension, and the research sample is limited to the floating population. These elements should be further studied by combining them with other data sources.

## Supplementary Information

**Additional file 1.**

## Data Availability

The datasets used in the current study is publicly available from China Migration Population Service Center [https://www.chinaldrk.org.cn].
