# Supplementary material for: How does air pollution affect urban settlement of the floating population in China? New evidence from a push-pull migration analysis
Source: BMC Public Health. 2021 Sep 17;21:1696. doi: 10.1186/s12889-021-11711-x (PMC8447744; doi:10.1186/s12889-021-11711-x)
Supplement: Supplementary file 1 — Additional file 1. [file 12889_2021_11711_MOESM1_ESM.docx]

**How does air pollution affect urban settlement of the floating population in China? Evidence from a push-pull migration analysis**

## Appendix

**Supplementary Table I**

Results from the heterogeneity test-Gender & Age

|  | Gender | |  | Age | | |
| --- | --- | --- | --- | --- | --- | --- |
|  | Female | Male |  | 15-29 | 30-44 | 45 above |
| Staywilling |  |  |  |  |  |  |
| PM2.5 | -0.042 | -0.132^***^ |  | 0.008 | -0.167^***^ | -0.085*** |
|  | (-1.39) | (-4.77) |  | (0.20) | (-5.34) | (-2.14) |
| City variables | control | control |  | control | control | control |
| Individual variables | control | control |  | control | control | control |
| _cons | 0.637^***^ | 0.921^***^ |  | 0.670^***^ | 0.952^***^ | 1.053^***^ |
|  | (5.74) | (8.94) |  | (5.14) | (8.42) | (7.25) |
|  |  |  |  |  |  |  |
| Pseudo R2 | 0.062 | 0.051 |  | 0.061 | 0.062 | 0.040 |
| Log likelihood | -23892.08 | -26368.21 |  | -16692.16 | -21182.88 | -12234.57 |
| N | 55837 | 60446 |  | 37757 | 52937 | 25589 |
| Notes: t statistics in parentheses  ** p<0.05, ** p<0.01, *** p<0.001* | | | | | | |

**Supplementary Table II**

Results from the heterogeneity test-Education

|  | Education | | | |
| --- | --- | --- | --- | --- |
|  | Less than primary | Junior school | Senior school | College above |
| Staywilling |  |  |  |  |
| PM2.5 | -0.069 | -0. 097^***^ | -0.169^***^ | 0.040 |
|  | (-1.50) | (-3.25) | (-3.80) | (0.66) |
| City variables | control | control | control | control |
| Individual variables | control | control | control | control |
| _cons | 1.127*** | 1.189^***^ | 1.161^***^ | 1.063^***^ |
|  | (5.55) | (12.53) | (8.72) | (6.02) |
| Pseudo R2 | 0.030 | 0.038 | 0.064 | 0.077 |
| Log likelihood | -9377.65 | -23582.01 | -10305.14 | -6909.32 |
| N | 17966 | 51137 | 25501 | 21679 |
| Notes: t statistics in parentheses  ** p<0.05, ** p<0.01, *** p<0.001* | | | | |

**Supplementary Table III**

Results from heterogeneity tests-hukou & Migration range

|  | *Hukou* | |  | Range | |
| --- | --- | --- | --- | --- | --- |
|  | (1) Urban | (2) Rural |  | (3) Inter-provincial | (4) Intra-provincial |
| Staywilling |  |  |  |  |  |
| PM2.5 | 0.028 | -0.126^***^ |  | -0.090^***^ | -0.127^*^ |
|  | (0.60) | (-5.51) |  | (-4.02) | (-2.35) |
| City variables | control | control |  | control | control |
| Individual variables | control | control |  | control | control |
| _cons | 0.677^***^ | 0.701^***^ |  | 0.737^***^ | 0.983^***^ |
|  | (4.29) | (9.63) |  | (8.66) | (8.17) |
| Pseudo R2 | 0.072 | 0.047 |  | 0.059 | 0.051 |
| Log likelihood | -9172.31 | -41093.68 |  | -31148.57 | -19094.92 |
| N | 25919 | 90364 |  | 71177 | 45106 |
| Notes: t statistics in parentheses  ** p<0.05, ** p<0.01, *** p<0.001* | | | | | |

**Supplementary Table IV**

Results from heterogeneity test-Monthly household income

|  | Income | | | |
| --- | --- | --- | --- | --- |
|  | Less than 4000 | 4000-6000 | 6000-8000 | 8000 and above |
| Staywilling |  |  |  |  |
| PM2.5 | -0.048 | -0.080^**^ | -0.143^**^ | -0.106^**^ |
|  | (-1.12) | (-2.17) | (-3.19) | (-2.64) |
| City variables | control | control | control | control |
| Individual variables | control | control | control | control |
| _cons | 0.208 | 0.713^***^ | 0.311^*^ | 0.288^*^ |
|  | (1.67) | (6.12) | (2.00) | (1.98) |
| Pseudo R2 | 0.035 | 0.030 | 0.035 | 0.053 |
| Log likelihood | -10976.081 | -14556.48 | -10440.52 | -14208.06 |
| N | 20067 | 29996 | 24263 | 41952 |
| Notes: t statistics in parentheses  ** p<0.05, ** p<0.01, *** p<0.001* | | | | |

**Supplementary Table V**

Results from heterogeneity test-Housing

|  | House | | |
| --- | --- | --- | --- |
|  | (1) Free housing | (2) Rent | (3) Self-purchased |
| Staywilling |  |  |  |
| PM2.5 | -0.135** | -0.103*** | -0.035 |
|  | (-2.89) | (-4.15) | (-0.61) |
| City variables | control | control | control |
| Individual variables | control | control | control |
| _cons | 0.577^***^ | 0.892*** | 1.498*** |
|  | (3.68) | (10.86) | (7.48) |
| Pseudo R2 | 0.030 | 0.035 | 0.049 |
| Log likelihood | -8875.83 | -33136.14 | -8165.69 |
| N | 17098 | 69883 | 29302 |
| Notes: t statistics in parentheses  ** p<0.05, ** p<0.01, *** p<0.001* | | | |

**Supplementary Table VI**

Results from heterogeneity tests-Rural contracted land & Homestead

|  | Contracted | | Homestead | |
| --- | --- | --- | --- | --- |
|  | (1) Contracted | (2) No contracted | (3) Homestead | (4) No homestead |
| Staywilling |  |  |  |  |
| PM2.5 | -0.120^***^ | -0.127^**^ | -0.045 | -0.163^***^ |
|  | (-3.64) | (-4.08) | (-1.06) | (-6.11) |
| City variables | control | control | control | control |
| Individual variables | control | control | control | control |
| _cons | 0.895^***^ | 1.175^***^ | 0.605^***^ | 1.134^***^ |
|  | (7.69) | (9.96) | (5.72) | (11.43) |
| Pseudo R2 | 0.056 | 0.045 | 0.065 | 0.045 |
| Log likelihood | -20138.14 | -22842.13 | -11918.61 | -31066.52 |
| N | 44051 | 51973 | 27214 | 68810 |
| Notes: t statistics in parentheses  ** p<0.05, ** p<0.01, *** p<0.001* | | | | |
